# Gender-related factors affecting access to TB services and treatment outcomes in the Philippines

**DOI:** 10.5588/pha.23.0021

**Published:** 2023-09-21

**Authors:** E. C. Mendoza-Hisey, A. Dier, N. V. Marquez, L. V. Bumanglag, S. B. A. Cadiao, S. F. Guirgis

**Affiliations:** FHI 360 Philippine Country Office, Makati, The Philippines

**Keywords:** gender-diverse persons, gender analysis, infectious disease, tuberculosis

## Abstract

**SETTING:**

The Philippines is one of the countries with the highest TB burdens. While TB affects men and women differently, studies also show that gender affects people’s experience of and access to healthcare. Men and women have usually assigned roles and responsibilities that affect their decisions and health-seeking behaviour.

**OBJECTIVE:**

The gender analysis aimed to examine the relationship between gender and access to TB services and treatment outcomes according to five domains: cultural norms and beliefs; patterns of power and decision-making; gender roles and responsibilities; access to resources; laws and policies.

**DESIGN:**

The team conducted 19 in-depth interviews and five focus group discussions with project staff, TB coordinators from healthcare facilities, representatives from the private and informal business sector and representatives from the Philippine Department of Health from August to November 2019.

**RESULTS:**

Study findings indicated that men faced greater limitations than women in terms of accessing TB resources and services, which highlight the differences between genders in relation to health-seeking behaviours and ability to access healthcare.

**CONCLUSION:**

This demonstrates the importance of integrating a gender lens into the service provision set up, from screening to treating and monitoring, to ensure equitable health benefits for men, women, transgender and gender-diverse persons.

## Abstract (fr)

**CADRE:**

Les Philippines font partie des pays ayant les plus lourds fardeaux liés à la TB. Bien que la TB affecte différemment les hommes et les femmes, des études montrent également que le genre influence l’expérience et l’accès aux soins de santé. Les hommes et les femmes ont généralement des rôles et des responsabilités assignés qui influencent leurs décisions et leur comportement en matière de recherche de soins de santé.

**OBJECTIF:**

L’analyse de genre visait à examiner la relation entre le genre et l’accès aux services de lutte contre la TB et les résultats du traitement selon cinq domaines : les normes et croyances culturelles ; les modèles de pouvoir et de prise de décision ; les rôles et responsabilités liés au genre ; l’accès aux ressources ; les lois et les politiques.

**MÉTHODE:**

L’équipe a réalisé 19 entretiens approfondis et cinq groupes de discussion avec le personnel du projet, les coordinateurs de la lutte contre la TB dans les établissements de santé, des représentants du secteur privé et informel, ainsi que des représentants du Ministère de la Santé des Philippines, d’août à novembre 2019.

**RÉSULTATS:**

Les résultats de l’étude ont indiqué que les hommes étaient confrontés à des limitations plus importantes que les femmes en termes d’accès aux ressources et aux services de lutte contre la TB, mettant en évidence les différences entre les genres en ce qui concerne les comportements en matière de recherche de soins de santé et la capacité d’accéder aux soins de santé.

**CONCLUSION:**

Cela démontre l’importance d’intégrer une perspective de genre dans la mise en place des services, de la détection au traitement et au suivi, afin de garantir des avantages sanitaires équitables pour les hommes, les femmes, les personnes transgenres et de genre diversifié.

Prior to COVID-19, TB was the leading cause of death worldwide from a single infectious agent, and is likely to again assume this role in the near future.^1^ In 2021, approximately 10.6 million people developed the disease, 6.6 million of whom were men and 4 million were women. According to the Global Burden of Disease Study conducted in 2019, the prevalence of global TB deaths attributable to alcohol use, smoking and diabetes was respectively 4.27 (range: 3.69–5.02), 6.17 (range: 5.48–7.02), and 1.17 (range: 1.07–1.28) times higher among men than women.^2^ More deaths and incident cases of TB-HIV co-infection occurred among women than men worldwide,^2^ including transgender women in the African region.^3^

These figures indicate clear disparities in how TB impacts men and women globally. Studies also show that gender affects people’s experience of and access to healthcare.^4^–^8^ Gender is defined as a socially constructed set of rules, responsibilities, entitlements and behaviours associated with being a man, a woman or a gender-diverse individual, and the relationships ­between and among people according to these constructs.^9^

Gender is different from sex, which refers to the different biological and physiological characteristics of ­females, males and intersex persons, such as chromosomes, hormones and reproductive organs.^10^

Note that although sex is biological, it is not limited to the binary male-female reproductive organ classification. Chromosomal studies show that there are several X and Y combinations that cannot be classified under the male-female binary system. Gender diversity, on the other hand, refers to an umbrella term that is used to describe gender identities that demonstrate a diversity of expression beyond the binary framework.^11^ Gender identity refers to a person’s deeply felt, internal and individual experience of gender, which may or may not correspond to the person’s physiology or designated sex at birth:

Transgender – or trans for short – is an umbrella term for those whose gender differs from that which they were assigned at birth. This includes binary trans people (trans men and trans women) and non-binary trans people, who may use descriptors like gender-queer, bi-gender, a-gender, or gender-fluid.^12^

Studies conducted within national TB programmes (NTPs) from other countries demonstrate that men and women experience different barriers when accessing TB diagnostic and treatment services. For example, in Pakistan women experience challenges such as ­limited autonomy in household financial decision-making, prohibitions on unassisted travel, long travel times, lack of prioritisation of spending on women’s health, and inadequate numbers of female health providers.^6^ In Bangladesh, Nepal and Pakistan, TB has a negative impact on the marriage prospects of women.^13^ In India, men have more unfavourable treatment outcomes, including death, failed treatment and lost to follow-up than women.^7^,^14^ Gender differences in the impact of TB may also be due to differences in health-seeking behaviour between men and women.^4^,^8^

The Philippines is one of the high TB burden countries, and the fourth major contributor of people who developed TB in 2021. Incidence of TB is estimated to be higher in men than women (519,000 per year vs. 222,000 per year). This ratio of 2.34 male-to-female exceeds the ratio of reported incident cases in the 2022 WHO Global TB Report, which was only 2.0.^1^ This means that although more cases are reported among men, the country is still disproportionately missing many men who are estimated to have TB. In addition, challenges in accessing TB services also differ greatly for men, women and transgender persons.^15^ For transgender and gender-diverse persons, the most common barriers to healthcare use are stigma, socio-economic challenges and systemic issues such as lack of provider knowledge and denial of health care services by service providers.^16^,^17^ Gendered roles dictated by Filipino cultural norms such as men being the main income earner of the family can prevent men from seeking medical consult because they prioritise work over their own health. Although women have more time to visit health facilities, gendered roles and responsibilities, insufficient funds and economic dependence on husbands limit access to health facilities.^18^ Reduced access leads to delayed TB diagnosis and treatment, which can cause further spread of TB among contacts.

The Philippine National Tuberculosis Control Program (NTP) has historically focused on addressing barriers across the continuum of care using the WHO health systems framework.^19^ The possibility that gender-related barriers may affect access to TB services and treatment outcomes had not yet been explored. The USAID-funded TB Innovations and Health Systems Strengthening (TBIHSS) project therefore conducted a gender analysis to understand how gender may influence health-seeking behaviours and/or impact on access to TB services and make recommendations on how the NTP of the Philippines could adapt the design and delivery of their programmes to improve TB services and better meet the unique needs of different communities.

## METHODS/STUDY DESIGN

Information was collected between August and November 2019 through five focus group discussions (FGDs) and 19 in-depth interviews conducted among pre-selected TB healthcare workers from rural health units located in project sites; health administrators; representatives from informal sector workers; staff from other USAID implementing partners; and government officials in Metro Manila, Central Luzon and Calabarzon. These three regions are among the most populated and are also priority areas for TB programme interventions for both the TBIHSS project and the Philippine government.

The gender analysis was guided by the five domains described in Chapter 205 of USAID’s Automated Directives Systems: access to resources, laws and policies, cultural norms and beliefs, patterns of power and decision-making, and gender roles and responsibilities.^20^ The Automated Directives Systems provides a framework to better understand the challenges and barriers that men, women and their families face in accessing TB services when affected by TB disease. This framework was used as the basis for the semi-structured question guides for both the FGDs and in-depth interviews. The question guides were pre-tested among project staff and revised based on feedback received (guides are available upon request). Interviewer/data collectors were trained on how to conduct the FGDs and interviews. Informed consent was obtained from participants before each interview or FGD was conducted. The interviews and FGDs were conducted in Filipino, recorded, directly transcribed and translated into English. Interview data were categorised into emergent themes using an inductive approach. The analysis was approved by the FHI 360 Office of International Research Ethics, Durham, NC, USA, on 16 July 2019.

## RESULTS

### Cultural norms and beliefs, patterns of power and decision-making and gender roles and responsibilities

Because patterns of power, decision-making, and gender roles and responsibilities are closely tied to cultural norms and beliefs and the patriarchally driven Filipino society, the findings for these three domains are presented collectively.

Patterns of power and decision-making in the household affect access to healthcare services and decisions in Filipino households and are usually made by men (fathers/husbands) as dictated by the country’s patriarchal culture. Furthermore, due to their predominant role as primary income earners within families, men’s gendered responsibilities significantly influenced their decision-making process regarding seeking healthcare services. Several participants mentioned that ‘men prefer to work…despite their health conditions than to take a rest because of TB.’ As government health centres and rural health units are often only open during working hours (8:00 am to 5:00 pm), it was reported that men were reluctant to take time off work to get tested for TB. This was especially true for labourers and informal sector workers who would lose income for missed time.

The word ‘macho’ was often used to describe men’s resistance to seeking healthcare and getting tested for TB. Macho is defined in the Oxford dictionary as being ‘aggressively proud of one’s masculinity.’ The macho image of men in the Philippines does not encourage health-seeking behaviour. Men consider themselves ‘tough’ and do not want to appear weak or sick, which can lead to denial of symptoms and failure to seek or follow through with treatment or accept a diagnosis. As a result of these gendered social norms and beliefs, men tend to wait until they are very ill, and their symptoms are quite severe before seeking treatment. This delay can lead to further TB transmission.

Other gender roles that impact men’s health and risk of TB infection include working in higher-risk occupations such as construction, mining and driving tricycles, which can compromise their respiratory health. Men are also more likely to smoke, drink alcohol or be incarcerated, and are therefore more vulnerable to TB.^21^–^24^ Women, on the other hand, are usually assigned to childcare and housekeeping roles. Although this set up allows them to have time to visit the health centres, they face financial challenges and are dependent on spousal income for healthcare.

It was noted that stigma related to TB diagnosis was often felt more acutely by men, which may be related to maintaining gendered social norms. Interviewees reported some cases where both men and women had been known to hide a TB diagnosis from their communities. Men were sometimes reluctant to seek treatment in their local health centre and would go further afield or pay for private healthcare to avoid recognition, which adds to the patient’s financial burden. Transgender and gender-diverse persons affected by TB disease are often shunned by society, which may have a negative impact on their health-seeking behaviour. According to a respondent, ‘transgenders and gender diverse are considered outcasts or not socially accepted by some people here.’

### Access to and control over health resources and services

Our findings indicate that men face greater limitations than women when accessing TB services due to their work schedule and actual or fear of lost earnings, especially if they were daily wage earners. Access to TB services for men and women in the workplace varied between the formal and informal sector. The formal sector has access to private medical insurance, which allows access to private hospitals and clinics at any time of the day, whereas those working in the informal sector only have access to local clinics with limited hours.

Although men were more likely to be earners in the family, women often handled household finances and budgets but needed permission from their husbands on how to spend the income earned. This is common even among couples where women had achieved higher education.^25^

### Laws, policies, strategies, and institutional context

The NTP is primarily guided and informed by the TB Law, Philippine Strategic TB Elimination Plan Phase 1 (PhilSTEP1) and the Filipino Manual of Procedures (MOP). The TB Law, also known as Republic Act No. 10767, established a comprehensive Philippine plan of action to eliminate TB as a public health problem. The PhilSTEP1 was rolled out in 2018 to guide TB programme managers nationwide on how to contribute to the national objectives. The MOP, on the other hand, was developed by the NTP to standardise TB management protocols and procedures across different health facilities in the country. These policies and guidelines addressed gender issues very briefly or not at all.^26^–^28^

Participants frequently referred to the MOP, which seemed to be strictly followed. The MOP refers to treating all genders ‘the same or equally,’ but does not acknowledge gender differences when managing symptoms in persons affected by TB disease.^27^ Equal treatment for all genders, including transgender and gender-diverse populations, is considered a non-discriminatory approach, equivalent to a patient-centred care approach.

In the past year, NTP staff attended a gender and development (GAD) training course at the Philippine Department of Health Central Office. However, the NTP did not have the budget required to implement any adaptations to existing GAD activities or to initiate new GAD activities. In relation to the existing activities, GAD initiatives primarily focus on women and children and there are no initiatives targeting men, transgender or gender-diverse persons. In addition, GAD policy implementation was reported to be poorly monitored, and some offices use the mandatory 5% funding for activities and projects completely unrelated to gender and development.

PhilSTEP1 includes a gender indicator but does not provide guidelines on how to measure gender-responsive programmes.^26^ However, participants reported awareness of gender disparities among persons affected with TB disease, as the NTP is encouraging strategies to address gaps and missing cases by advising regions and health facilities to focus on men at high risk through active case-finding and screening (i.e., tricycle drivers, smokers and prison inmates).

The TB ordinance implemented in Central Luzon is an example of a gender-blind policy, as the unique gender roles and different needs of men, women, and transgender and gender-diverse persons were not taken into account when addressing TB. Some participants were aware of the Philippine Commission on Women’s (PCW’s) GAD plan and gender-responsive local government, but neither initiative seemed to be applied directly to NTP activities at the regional level and local government units.

Both men and women working in the formal sectors fear losing their jobs if they are diagnosed with TB and their status is disclosed. Lack of confidentiality, failure to safeguard patient data and a lack of awareness or implementation of policies relating to TB and workplace discrimination can lead to ongoing stigmatisation.

## DISCUSSION

### Key recommendations

This gender analysis identified numerous avenues for further exploration and possible changes to TB care in the Philippines. The study was undertaken specifically to identify the ways the NTP could adapt their programme design and delivery, and the following are our key recommendations: 1) the NTP could improve data collection of all TB services to include gender data, so that change at the patient level can be measured as gender-related improvements to the NTP programmes are initiated; 2) NTP could look to applying an equity lens rather than an equality lens when identifying focus communities; and 3) as participants in the study predominantly adopted a gender binary position, the NTP could conduct a study to understand what impact this may have on creating barriers for transgender and gender-diverse population seeking TB services. The Table gives some of the recommendations corresponding to the gaps and challenges identified under the different domains.

**TABLE:** Gaps and challenges identified in the gender analysis and their corresponding recommendations

Gap/challenge identified	Recommendation
Cultural norms and beliefs, patterns of power and decision-making, and gender roles and responsibilities	
Patterns of power and decision-making in the household affect access to health care services and decisions in a Filipino household are usually made by men (fathers/husbands) as dictated by the country’s patriarchal culture	To eliminate TB, behaviour change, and communication interventions should reach beyond interpersonal communication to community-wide campaigns through mass media to produce a transformative change in gendered cultural norms, beliefs, and behaviour^29^
Men’s gendered roles affected their decision whether to seek health care. Several participants mentioned that, ‘men prefer to work…despite their health conditions than to take a rest because of TB’	Key government agencies such as the Department of Education and DOH should work with the private sector to deliver key messages and advertising campaigns that not only provide information about TB and how it is transmitted, but also challenge and change traditional gender norms. For example, the TBIHSS project developed an online training module for private companies on how to implement TB programming in the workplace. This module was uploaded to the website of a TBIHSS local partner in the private sector, Employers Confederation of the Philippines. Gender-related content was integrated into this training module to raise awareness on gender differences and challenges faced by workers Since those most commonly affected by TB are within the working age group, providing social protection programmes to bolster economic security may be an important intervention to address socio-economic challenges faced by people affected by TB disease.^30^ Resolving social barriers and related health system obstacles should require attention and action by programme managers and policymakers^31^
The word ‘macho’ was often used to describe men’s resistance to seeking health care and getting tested for TB. Macho is defined in the Oxford dictionary as ‘aggressively proud of one’s masculinity.’ The macho image of men in the ­Philippines does not encourage health-seeking behaviour. Men consider themselves ‘tough’ and do not want to appear weak or sick, which can lead to denial of symptoms and failure to seek or follow through with treatment or accept a diagnosis	
Access to and control over health resources and services	
Binary perception of many TB programme managers and health workers does not address the unique needs of transgender and gender diverse individuals, resulting in a dearth of data on this population in this gender analysis	Training of all staff to understand how their actions impact accessibility of health services. Barangay health workers should reach out to individuals in their catchment areas through community-based mechanisms to reduce feelings of isolation, inform community members about available government services, and encourage proactive health seeking by people of all genders
Men are at higher risk for TB	Health care facilities should explore alternative clinic schedules to accommodate work schedules, especially for male-dominated occupations (e.g., construction, mining, and jeepney/tricycle drivers), as these jobs usually do not offer paid leave of absence
Laws, policies, strategies, and institutional context	
The Philippine MOP did not address gender differences in finding, preventing, or curing TB	National TB strategic plans must include activities that address gendered barriers in accessing health care services. Interventions are needed to actively communicate, especially to men, the importance of early diagnosis and treatment. Health policies and programmes need to incorporate gendered power dynamics when it comes to decisions regarding TB and health care. This can be achieved by changing norms of power dynamics that currently may negatively impact people’s health
The TB ordinance implemented in Central Luzon is another example of a gender-blind policy, as it did not consider the unique gender roles and different needs of men, women, and transgender and gender diverse persons when addressing TB	Policymakers and programme managers should create policies and strategies that would enable men, women, and transgender and gender diverse people to influence and act on their health outcomes Health care workers (e.g., doctors, nurses, and barangay health workers) play a key role in implementing policies at the community and health care facility levels through community mobilisation and communicating health care messages. National level rules and guidelines should be cascaded to the community level, with local municipalities ensuring that budgets are sufficiently allocated for health care workers to implement these policies and close the policy-to-practice gap. To assess the impact of policies and strategies, monitoring and evaluation plans should have gender-sensitive indicators in addition to sex-disaggregated data. A detailed description on how to collect such data would be helpful for TB programme managers
Both men and women working in the formal sectors fear losing their jobs if they are diagnosed with TB and their status is disclosed	As part of governments’ economic and health agendas, policies should enhance the capacity of women and transgender and gender diverse people to make decisions to improve their health without being hindered by their economic situation or patriarchal norms
Lack of confidentiality, failure to safeguard patient data, and a lack of awareness or implementation of policies relating to TB and workplace discrimination can lead to ongoing stigmatization	The Department of Health and Department of Labor and Employment influence how gender dynamics of TB are prioritised and addressed in laws, policies, and strategies. These government agencies are responsible for creating advisories and training to guide both public and private entities on new laws and policies, and these advisories and training should be included in onboarding requirements for new employees^32^

DOH = Department of Health; TBIHSS = TB Innovations and Health Systems Strengthening; MOP = Manual of Procedures.

### Limitations

The authors acknowledge the following limitations of this gender analysis. Data on transgender populations and gender-diverse individuals were scarce, potentially due to the binary perception of most participants. No patients were included in the research. Although the data provided by health workers were reliable, health workers cannot speak on behalf of focus communities, especially indigenous, transgender and gender-diverse people. While this means that this gender analysis does not provide a detailed analysis, it does open avenues of opportunity for further research aimed at identifying gender-specific recommendations for the different groups identified.

## CONCLUSIONS

Although the study yielded limited data on experiences of transgender and gender-diverse communities accessing TB services, this reflected the typical perceptions held by participants and the dominance of gender norms. The subsequent actions entail engaging individuals from marginalised communities to gain insight into the obstacles they encounter when seeking healthcare, and enhancing healthcare programmes accordingly. Our findings highlight the differences between genders in relation to health-seeking behaviours and ability to access healthcare. This demonstrates the importance of integrating a gender lens into the service provision set up to ensure equitable health benefits for men, women, transgender and gender-diverse persons.
